# Meta-analysis of randomized controlled trials assessing the efficacy of *nigella sativa* supplementation for allergic rhinitis treatment

**DOI:** 10.3389/fphar.2024.1417013

**Published:** 2024-09-20

**Authors:** Yuxiao He, Xiaoyan Hu, Lanyin Chang, Shuang Liu, Liangge Lv, Gang Qin, Liang Jiang

**Affiliations:** ^1^ Department of Ophthalmology and Otolaryngology, Yanjiang District People’s Hospital of Ziyang City, Ziyang, Sichuan, China; ^2^ Department of Pathogen Biology, School of Basic Medicine, Southwest Medical University, Luzhou, Sichuan, China; ^3^ Department of Otolaryngology, Zigong Third People’s Hospital, Zigong, Sichuan, China; ^4^ Department of Otolaryngology, Affiliated Hospital of Southwest Medical University, Luzhou, Sichuan, China

**Keywords:** Nigella sativa, allergic rhinitis, randomized controlled trial, *Nigella* spp, *Nigella* glandulina

## Abstract

**Background:**

Demonstrating the positive effects of *Nigella sativa* supplementation (*Nigella* spp) on Allergic Rhinitis (AR) treatment is challenging. To investigate the role of *Nigella* spp in managing AR, a comprehensive review through systematic reviews and meta-analyses was conducted.

**Purpose:**

To carry out a meta-analysis of clinical trials that used *Nigella spp* in treating AR based on current data.

**Study Design:**

A meta-analysis of randomized controlled trials (RCTs) was performed.

**Methods:**

Various databases, including PubMed, Web of Science, Embase, Science Direct, Springer Link and the Cochrane Library, were searched until October 2023 to obtain RCTs assessing impact of *Nigella* spp in the control of AR. The current meta-analysis was carried out with a random-effects model.

**Results:**

There were 8 studies enrolled, and our meta-analysis findings revealed that, relative to the control group, observation group exhibited the markedly increased total effective rate for allergic rhinitis treatment (odds ratio [OR] = 4.24, 95% confidence interval [CI] (2.57, 7.27), and *p* < 0.00001); three studies showed that the effect of *Nigella* spp for nasal symptoms treatment among patients with allergic rhinitis was superior in observation group to control group [mean difference = −2.60, 95% CI (−2.82, −2.38), *p* < 0.00001]; adverse effects occurred in five studies, all of which were transient, did not require medical intervention, and were not statistically significant between the two groups [OR = 1.01, 95% CI (0.59, 1.73), *p* = 0.98].

**Conclusion:**

The observation group demonstrated relative safety and had an enhanced effect on allergic rhinitis treatment and total nasal symptom improvement than the control group. The inclusion of fewer studies and the lower quality of trial design might affect the stability of the results. However, the evidence-based findings that *Nigella* spp for allergic rhinitis treatment is more accurate should be validated in future large-scale, multicenter, and well-designed RCTs.

## 1 Introduction

Nigella sativa, commonly known as black cumin, is an annual plant that belongs to the genus Nigella of the Ranunculaceae family. This plant originates from the Mediterranean region, where it has been cultivated for thousands of years. Nigella sativa has several common names. In English, it is often called black cumin or black caraway. Its flower is known as “love-in-a-mist” due to its unique appearance, and in Chinese, it is referred to as “Persian gem.” In India and Urdu-speaking regions, it is known as “Kalonji.” The triangular black seeds of Nigella sativa resemble onion seeds, black poppy seeds, and its leaves resemble those of coriander and fennel, making identification challenging. However, Nigella sativa belongs to the Ranunculaceae family and is not taxonomically related to these plants (Zhang and Zhang, 2014). Nigella sativa has been widely used in various cultures for both culinary and medicinal purposes. In traditional medicine, it has been employed for treating a range of ailments due to its supposed anti-inflammatory, antioxidant, and immune-boosting properties. Despite these uses, it is important to note that Nigella sativa contains toxic compounds, which has limited its medicinal applications historically (Otolaryngology, 2022).

In ancient Mongolian medicine, Nigella sativa supplementation (Nigella spp) was commonly used, but it has since been replaced by Nigella glandulifera. This shift is possibly due to the artificial cultivation of Nigella glandulifera in China, ensuring a steady supply and reducing dependence on imports. Despite Nigella sativa’s better medicinal effects, its reliance on imports led to its gradual replacement in Mongolian medicine (Bauchau and Durham, 2004; Tungsukruthai et al., 2018; Gul et al., 2022). Allergic rhinitis is a non-infectious chronic inflammatory disorder of the nasal mucosa, primarily triggered by immunoglobulin E following allergen exposure. Clinical manifestations include sneezing, nasal itching, nasal congestion, and runny nose. This condition affects 10%–20% of the global population, posing a significant health problem (Ali and Blunden, 2003). Recent studies indicate that the incidence of allergic rhinitis ranges from 11.1% to 17.6% globally, and 23%–30% in China and Europe, respectively (Zhang and Zhang, 2014; Boskabady et al., 2004).

Current clinical treatment for allergic rhinitis follows the “four-in-one” principle: avoiding allergens, standardizing drug therapy, allergen-specific immunotherapy, and enhancing patient education (Huffman, 2003). However, these treatments often have numerous side effects, long treatment cycles, and lack long-term efficacy. Several herbal remedies have been explored in human or animal models for treating allergic rhinitis (Moher et al., 2009).

Nigella sativa has been used for over 2,000 years to treat various disorders and has shown potential efficacy against allergic rhinitis in both human and animal studies (Higgins and Green, 2011; Zhao et al., 2020). Although alfalfa seeds have demonstrated anti-inflammatory and antihistamine effects (Jadad et al., 1996; Kjaergard et al., 2001), evidence supporting the routine use of black cumin supplements for treating allergic rhinitis is lacking. Research analyzing the effects of Nigella spp on allergic rhinitis has yielded inconsistent findings (Higgins and Thompson, 2002; Abdulghani Mohamed et al., 2012).

The present systematic review and meta-analysis of randomized controlled trials (RCTs) focused on evaluating effect of *Nigella* spp in allergic rhinitis patients.

## 2 Materials and methods

This present work was carried out following Cochrane Handbook for Systematic Reviews on Interventions guidelines and preferred reporting items for systematic reviews and meta-analyse (Alsamarai et al., 2014).

## 3 Ethical considerations

Since our analysis was conducted on existing study data, informed patient consent or ethical approval was not required for this meta-analysis.

## 4 Study screening

1. Literature inclusion criteria
(1) Study type: All clinical RCTs on the treatment of allergic rhinitis with *Nigella* spp, regardless of the publication type, time, and language.
(2) Study subjects: Patients diagnosed with allergic rhinitis regardless of gender, age, race, education, and economic status.

(3) Intervention type: The intervention type included *Nigella* spp as monotherapy or in combination with Western medicine for the observation group. The control group received only Western medicine, including loratadine tablets, assamine, ephedrine, dexamethasone, Claritin syrup, montelukast chewable tablets, mometasone furoate nasal spray, levocetirizine hydrochloride throat capsules, either alone or in combination.

(4) Outcome measures: primary indicator, total effective rate, and secondary indicator, total nasal symptoms and adverse reactions.
2. Literature exclusion criteria
(1) Nonclinical RCTs; (2) Duplicate publications; (3) Medical cases, doctors’ experience, reviews, and animal experiments, (4) studies where complete data were unavailable; (5) Studies on external treatment with traditional Chinese and Western medicine; (6) Studies that excluded patients diagnosed with allergic avoidance comorbidities; and (8) Studies with insufficient safety and efficacy data.
3. Selection criteria

Multiple databases, Embase, PubMed, Web of Science, Science Direct, Springer Link and the Cochrane Library, were searched till October 2023, using the following keywords: Rhinitis Allergic, Allergic Rhinitides, Rhinitides Allergic, Allergic Rhinitis and *Nigella sativa*, sativa Nigella, Cumin Black, Kalonji, Kalonjus, Black Cumin, Black Cumins, and Cumins Black. References from the obtained articles were manually searched several times for including more qualified articles. These following studies were included: (1) RCTs, (2) allergic rhinitis patients, and (3) Intervention with *Nigella* spp compared with a placebo.

## 5 Data collection and outcome measurement

### 5.1 Data collection

The basic data, including the first author name, case number, age, females, and detailed methods for both groups from the included articles were obtained via two reviewers. Disagreements were settled through consensus. Data were obtained from corresponding authors if needed. Our primary and second outcomes were total effective rate and total nasal symptoms/adverse reactions separately.

Based on the electronic database search strategy outlined above, two researchers conducted searches in both Chinese and English electronic databases. They used EndNote X7 software to identify and remove duplicate studies, integrated the search results from the different databases, created an information database, and downloaded the full texts of the relevant studies. Subsequently, two researchers independently performed preliminary screening and extracted data according to a pre-defined table. They cross-checked and reviewed the extracted data, recorded the reasons for excluding each study, and consulted third-party experts to resolve differing opinions and reach a final decision. The data extraction encompassed fundamental details from the included studies (e.g., first author and publication year), pertinent information about the experimental and control groups (such as case numbers, intervention measures, and Jada fraction), and the study design along with quality assessment data (including randomization methods, blinding procedures, allocation concealment, completeness of outcome data, selective reporting, and other sources of bias).

Literature screening and data extraction selection. Two independent investigators screened all searched titles, abstracts, as well as full-texts. Differences between them were settled through discussions with a third-party reviewer. Data from each article was recorded on a predesigned Excel sheet, including author information, year of publication, use of random allocation method, total number of cases, number of observation and control groups, treatment method for observation and control group, effective rates for observation and control groups, course of treatment, and adverse reactions. Quality evaluation of the literature: study quality was assessed in accordance with the Cochrane Review Manual, mainly concerning random allocation method, concealment of allocation, participant blinding, outcome assessment blinding, the sufficient outcome data, selective reporting, or additional bias sources, and the quality of each literature was evaluated through three levels: “high risk,” ”low risk.” and “uncertain risk.”

### 5.2 Outcome measures

Statistical analyses were performed using RevMan 5.4 software. The odds ratio (OR) was used to represent dichotomous outcomes, while mean difference (MD) was used for continuous outcomes. All estimates were presented with 95% confidence intervals (CIs). I2 values were adopted for assessing heterogeneity. If I2 was <50%, statistical analysis will be performed using a fixed-effect model; otherwise, statistical analyses will be performed with the random-effects model, and heterogeneity sources will be explained.

## 6 Quality assessment of the literature

### 6.1 Literature search analysis

**FIGURE 1:**
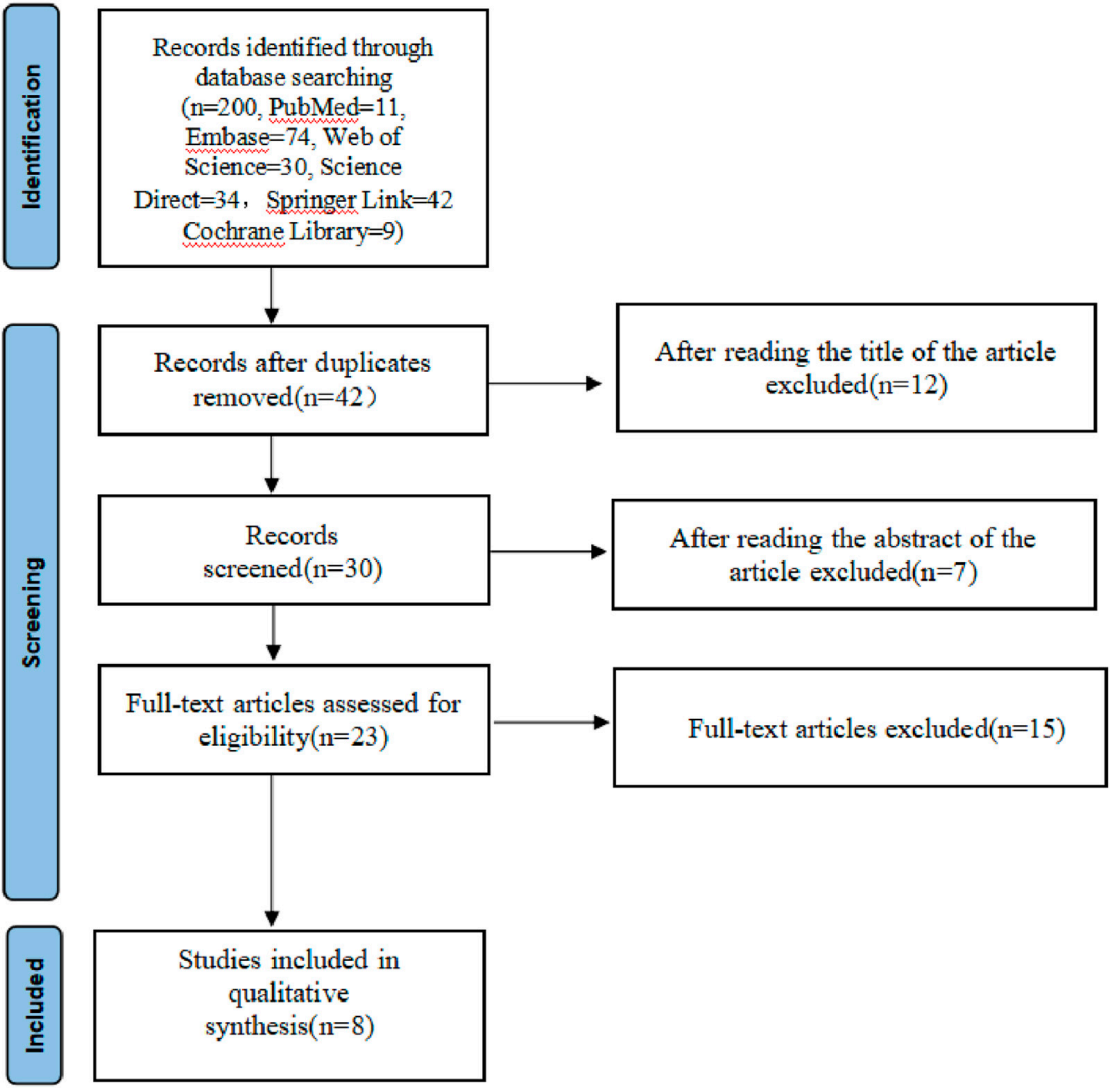
Flowchart showing the screening and selection of this study.

A total of 200 studies were reviewed, including 11 in PubMed, 74 in Embase, 30 in Web of Science, 34 in Science Direct, 42 in Springer Link and 9 in the Cochrane Library. NoteExpress 3.6.0.923 software was used to delete the 33 duplicate articles. Titles and abstracts of those rest 91 articles were further checked, of which 18 irrelevant articles were deleted. The remaining articles were downloaded according to the inclusion criteria, and of these, 15 articles were read, among which, 8 failed to meet the criteria, resulting in the enrollment of 8 studies (Figure 1).

### 6.2 Basic features of qualified literature

In those seven enrolled studies (Nikakhlagh et al., 2011; Ansari et al., 2009; Işik et al., 2010; Daghistani, 2005; Kalus et al., 2003; Liao and Bai, 2022), the treatments used for the Nigella and control groups can be observed from Table 1.

**TABLE 1:** Features of the enrolled articles.

Author (year)	*Nigella* group				Control group				Jada fraction
	Number	age	Woman (n)	way	Number	age	Woman (n)	way	
Alsamarai, A (2014)	38	25.1 ± 8.3	-	(Each drop container is about 15 mL, and the patient takes 2 drops (one drop in each nostril) thrice/day for 6 weeks)	30	25.1 ± 8.3	-	placebo	4
Nikakhlagh, S (2011)	30	29.8 ± 6.8	-	500uL/kg alfalfa Neisser grass was taken orally for 30 days	29	29.8 ± 6.8	-	placebo	4
Ansari, M. (2009)	23	31.6 ± 1.9	-	250 mg/day of alfalfa orally for 2 weeks	22	31.6 ± 1.9	-	placebo	4
Işik, H (2008)	12	34	-	2 g/day, orally, for 30 days	8	23	-	placebo	3
Wadiah S (2005)	50	38 ± 8.9	-	100 mg of alfalfa (whole seed) once a day at bedtime for 4 weeks	16	38 ± 8.9	-	placebo	4
Kalus, U (2003)	20	40 ± 13.4	12	Black cumin oil 40–80 mg/kg/day Lasts 6–8 weeks	22	11.5 ± 2.9	-	placebo	3
Liao, C (2022)	39	33.51 ± 10.70	23	With Urite-3 (including 3 g of black cumin vulgaris, 1 g of licorice, and 1 g of Xiaoyinxiang), daily dose, 1 pack each time, thrice/day, 15 days for 1 course of treatment	39	30.74 ± 7.27	23	placebo	3
Ridha C (2023)	21	31	-	2 gr for 28 days	21	34	-	placebo	3

### 6.3 Study quality assessment

**FIGURE 2:**
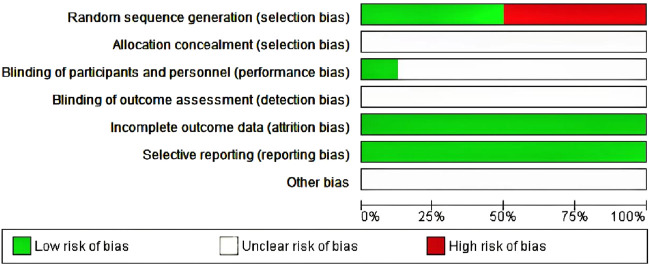
Study quality evaluation results.

The Jadad scale, including three assessment elements, namely, randomization (0–2 points), blinding (0–2 points), and withdrawal (0–1 point), was adopted for assessing the RCT method quality (Nikakhlagh et al., 2011). If each element was appropriately discussed and mentioned in the original text, a point was assigned to those elements. The Jadad Scale scores were in the range of 0–5. Articles with Jadad scores ≤2 were low-quality, whereas those with Jadad scores ≥3 were high-quality (Işik et al., 2010; Daghistani, 2005). Of the seven included studies, three used a specific randomized scheme of random number tables, whereas the other four did not mention a specific randomization scheme; the specific allocation concealment method participant blinding, outcome assessment blinding were not mentioned. No outcome data were missing, no selective reporting was found, and other sources of bias were unknown (Figure 2).

## 7 Results of meta-analysis

### 7.1 Total effective rate

**FIGURE 3:**
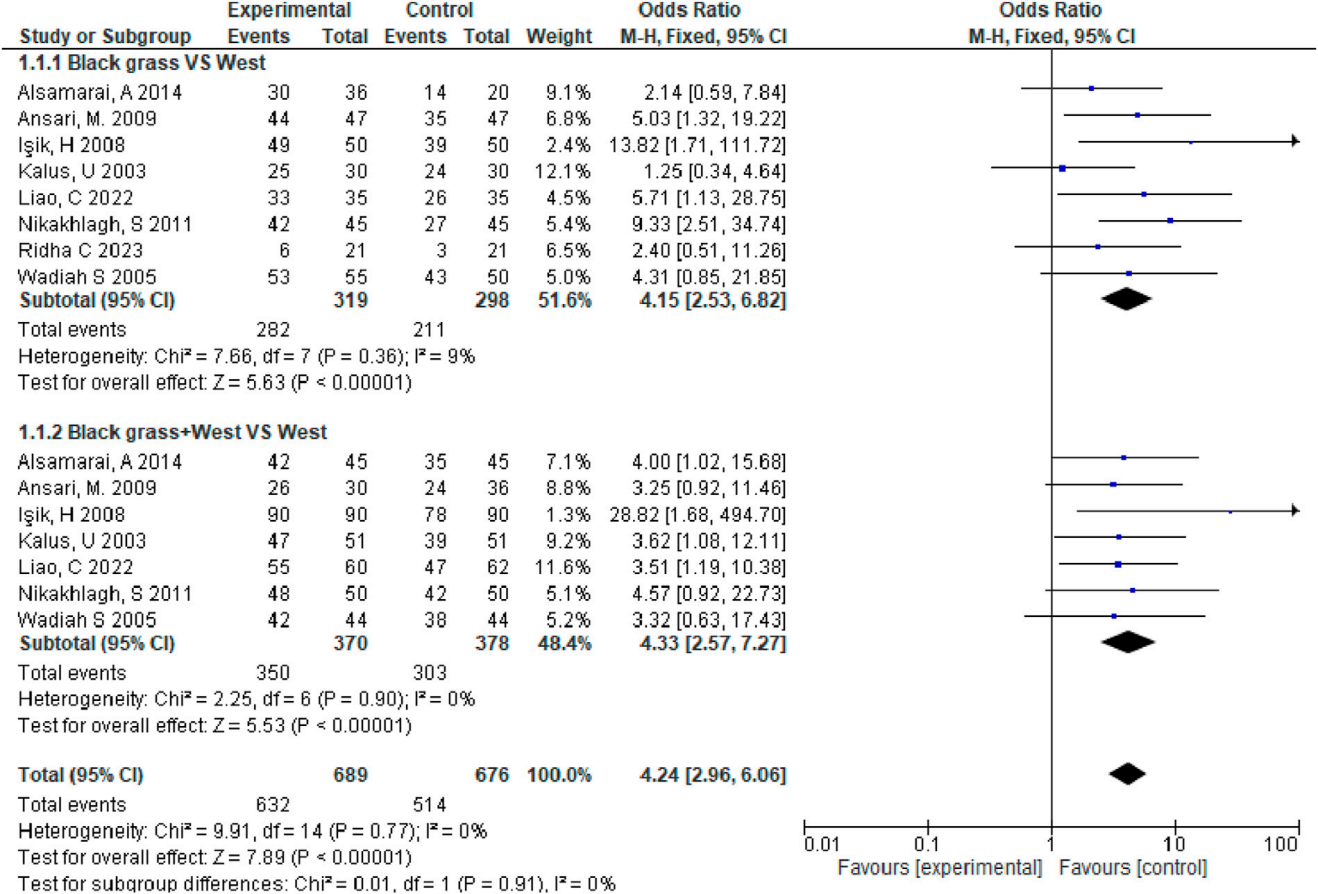
Forest diagram of total effective rates.

Statistical heterogeneity (I2 = 0 < 50%) was not detected among the articles, so we selected a fixed-effect model. According to our analysis, relative to control group, observation group exhibited the markedly increased total effective rate for allergic rhinitis [OR = 4.24, 95% CI [2.57,7.27], *p* < 0.00001](Figure 3), which indicated that the treatment effect on allergic rhinitis patients in the experimental group was significantly better than that in the control group. With the administration of black seed, the patients’ therapeutic outcomes were markedly improved.

### 7.2 Total nasal symptoms

**FIGURE 4:**
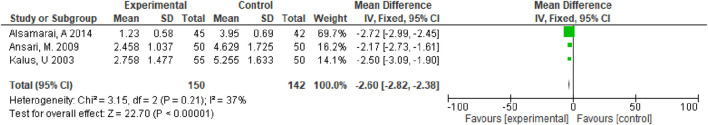
Total nasal symptoms.

Of the seven enrolled studies, three studies evaluated total nasal symptoms, all of which were Nigella *versus* Western medicines. As heterogeneity (I2 < 50%) was not detected, we applied a fixed-effect model. Based on our meta-analysis, relative to control group (Western medicine), observational group (black cumin) achieved a significantly superior effect on treating allergic rhinitis for relieving total nasal symptoms (MD = −2.60, 95% CI [−2.82, −2.38], *p* < 0.00001) (Figure 4).

### 7.3 Improvement in total symptoms, pruritus, sneezing, and runny nose

**FIGURE 5:**
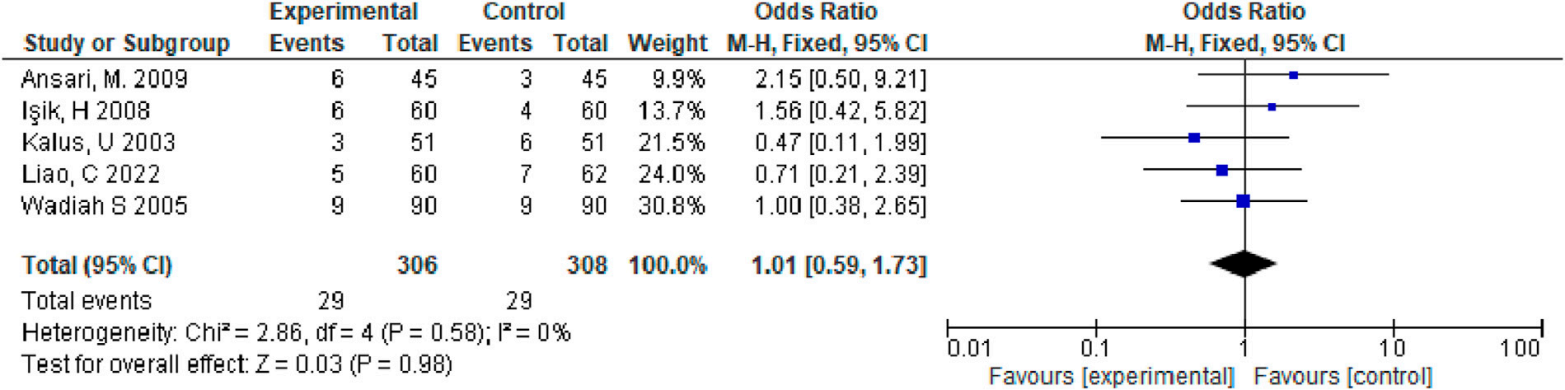
Forest diagram of adverse reactions.

We assessed standard ORs as well as 95% CIs of binary outcomes (improvement in total symptoms, pruritus, sneezing, and runny nose). I2 statistics revealed I2 >50%, suggesting obvious heterogeneity (Moher et al., 2009). Therefore, we applied the random-effects model in meta-analysis. We identified the likely sources of heterogeneity to obtain obvious heterogeneity. Meanwhile, sensitivity analyses were performed to determine influence of individual studies on total estimate by sequentially eliminating an article or conducting subgroup analyses. Overall, seven studies were included, and our meta-analysis revealed that relative to control group, observation group exhibited markedly increased total effective rate for treating allergic rhinitis (OR = 4.24, 95% CI [2.57,7.27], *p* < 0.00001). The above findings showed that the effect of *Nigella* spp remarkably increased in observation group compared with control group for treating total nasal symptoms among patients with allergic rhinitis (MD = −2.60, 95% CI [−2.82, −2.38], *p* < 0.00001). Adverse effects, such as nasal dryness, hoarseness, nausea, dry mouth, drowsiness, malaise, and stomach discomfort occurred in five studies. However, the adverse reactions were transient and did not require medical intervention. The adverse reaction rate was nonsignificant in observation *versus* control groups (OR = 1.01, 95% CI [0.59, 1.73], *p* = 0.98) (Figure 5), this meant that adverse reactions would not significantly change due to the alteration in the treatment method.

### 7.4 Publication bias

**FIGURE 6:**
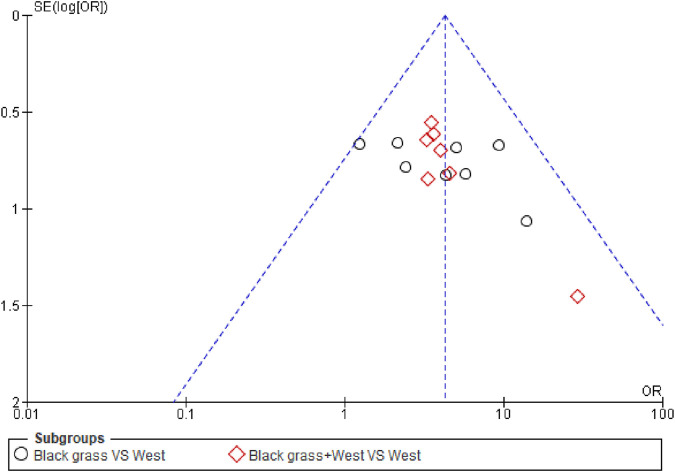
Funnel plot on publication bias.

The efficacy rate of the seven enrolled articles was evaluated for publication bias, and the funnel plot analysis showed asymmetrical scatter distribution on both sides, indicating the presence of publication bias in enrolled articles, as shown in (Figure 6).

## 8 Results

Figure 1 shows the search and screening criteria for this study. Table 1 outlines the baseline characteristics of the seven RCTs included in our meta-analysis, conducted between 2003 and 2014, with a combined sample size of 318 participants. The duration of Nigella spp administration ranged from 2 to 8 weeks. Two RCTs (Nikakhlagh et al., 2011; Ansari et al., 2009), reported improvement in total symptoms, while two others (Kalus et al., 2003; Liao and Bai, 2022) observed improvements in pruritus, sneezing, and runny nose. The overall effective rate for allergic rhinitis significantly increased in the observation group compared to the control group, with an odds ratio (OR) of 4.39 and 95% confidence interval (CI) of [3.09, 6.24], *p* < 0.00001 (Figure 3). Significant improvements in total nasal symptoms were also noted in the observation group treated with Nigella sativa, with a mean difference (MD) of −2.60 (95% CI [−2.82, −2.38], *p* < 0.00001) (Figure 4).

Adverse events were reported in five studies. The control group experienced nasal dryness (six reports), hoarseness (two), nausea (three), dry mouth (six), drowsiness (five), malaise (three), headaches (four), and gastric discomfort (two). The observation group reported similar adverse reactions but with slightly different frequencies: nasal dryness (seven), hoarseness (three), nausea (three), dry mouth (six), drowsiness (six), malaise (three), and gastric discomfort (one). These adverse reactions were transient and did not necessitate medical intervention, with no severe adverse effects reported (Figure 5). Despite the limited number of studies and potential issues with trial design quality, which may introduce publication bias as indicated by the funnel plot (Figure 6), the results of this meta-analysis are significant and suggest that Nigella sativa could be effective in treating allergic rhinitis. Future research should focus on larger, multicenter, and rigorously designed RCTs to confirm these findings.

## 9 Discussion

Allergic rhinitis is a chronic disorder, with current treatments primarily focusing on symptom alleviation during acute attacks using pharmacotherapy. Pharmacotherapy relies on Western drugs, such as antihistamines, anticholinergics, glucocorticoids, antileukotrienes, and decongestants (Liao and Bai, 2022; Wang et al., 2023). Recent advances in ethnic medicine, particularly Mongolian medicine, have shown promising results in treating allergic rhinitis. This includes therapies like Thirteen Flavor Safflower Secret Pills and Gu Ri Chong, which integrate Chinese and Western medical practices. *Nigella sativa*, the annual herbaceous plant, belongs to genus Nigella in the family Ranunculaceae, including three species: *Nigella* spp, *Nigella* glandulina, and *Nigella* nigvana, among which *Nigella* nigrena and *Nigella* glandulina have medicinal value. Nigella is known as “Hari Shu-Saila,” ”Ha Ri Yue-Zila,” or “Sera Naheb”in Mongolian medicine and was first recorded in the “Unmistakable Mongolian Medicine,” which belongs to the family *Nigella* spp. Mongolian doctors believe that Nigella has a harmonizing effect on the liver and spleen, and by regulating the turbidity and sublimation, adjusting the three roots of the human body, improving immunity, and is widely used in Mongolian medicine prescriptions, such as Zhenbao Pill, Eight Flavor Pomegranate Powder, Shunqi Thirteen Flavor Powder, and Black Cumin Seed Five Flavor Powder. (Liao et al., 2022; Liao and Bai, 2022). *Nigella sativa* extract significantly alleviates typical symptoms during an acute attack of allergic rhinitis, like nasal itching, nasal membrane congestion, runny nose, sneezing, and turbinate hypertrophy. It has an identical effect, similar to cetiril, in mitigating acute seasonal allergic rhinitis symptoms and does not cause adverse reactions. Licorice has been frequently applied in traditional Chinese medicine formulations, and its active ingredient glycyrrhizic acid has obvious anti-infective effects (Han and Chen, 2021). Glycyrrhizic acid, a metabolite of glycyrrhizic acid in the human body, has an adrenal corticosteroid-like structure, which can inhibit the production of leukotrienes and other inflammatory mediators and reduce the free release of arachidonic acid, thereby inhibiting the release of allergic substances, neutralizing allergens, and stabilizing cell membranes for preventing and treating allergic rhinitis. Xiaoyinxiang is rich in fatty oils, volatile oils, +< alcohols, glycols, alkaloids, and other active ingredients; it has effects against inflammation and analgesia, reducing tumor necrosis factor-α activity while decreasing inflammatory cell number through the regulation of interleukin (IL)-10 and IL-17 levels (Han and Liao, 2020).

Several studies have documented treating allergic rhinitis with *N. sativa* and demonstrated positive impacts on controlling allergic rhinitis, such as symptom scores, polymorphonuclear leukocytes function, immunoglobulin E in nasal lavage, and eosinophils in the nasal mucosa (Chen, 2020). Recent studies indicated that systemically using alfalfa extract effectively reduces symptoms of mild to moderate allergic rhinitis. Factors such as age, gender, and genetic predispositions can influence the efficacy of *N. sativa* in treating allergic rhinitis, highlighting the complexity of individual responses and the potential for varied outcomes. The use of alfalfa extract is noted for its simplicity and ease of dosage control, although its effectiveness requires further validation through rigorous scientific studies (Tian, 2014).

Nigella sativa was used both as a standalone treatment and in combination with other medications. When used in association, it was commonly combined with antihistamines or corticosteroids (Liao and Bai, 2022; Wang et al., 2023). The combination was aimed at enhancing therapeutic efficacy and reducing the required dose of conventional drugs. In the treatment of allergic rhinitis, Nigella Sativa (black seed) can be used both independently and in combination with other medications (Tian, 2014). When used independently, Nigella Sativa oil or extract helps alleviate the symptoms of allergic rhinitis through its anti-inflammatory, anti-allergic, and immunomodulatory properties (Ahmad A, 2013). In combination therapy, Nigella Sativa is often paired with antihistamines, nasal corticosteroids, and decongestants to enhance efficacy (Ahmad A, 2013). Among these, the most commonly used pharmacological category is antihistamines, as they directly target the symptoms of allergic reactions, providing rapid relief for patients. Nigella Sativa can enhance the effects of antihistamines, offering more comprehensive symptom management (Liao and Bai, 2022; Wang et al., 2023).

The proportion of improvement in allergic rhinitis symptoms after using Nigella Sativa varies across different studies (Han and Chen, 2021). The significant improvement in allergic rhinitis symptoms was reported by approximately 70% of patients after using Nigella Sativa oil for 8 weeks, as found by one study. Patients experienced reductions in nasal congestion, itching, sneezing, and runny nose, leading to overall better quality of life (Tian, 2014). Symptom relief was experienced by about 80% of patients after using a Nigella Sativa nasal spray for 4 weeks, as indicated by another study. These patients noted marked decreases in the frequency and severity of their allergic reactions, particularly during high pollen seasons (Tian, 2014). Over 60% efficacy in treating allergic rhinitis was demonstrated by a third study using Nigella Sativa extract (Ahmad A, 2013). Improvements included a reduction in nasal obstruction, fewer episodes of nasal itching, and a general decrease in the need for additional allergy medications. These studies highlight Nigella Sativa’s potential in providing substantial symptom relief for individuals suffering from allergic rhinitis. (Liao et al., 2022).

The safest and most effective dose of Nigella sativa was found to be around 500–1,000 mg per day, depending on the form of the supplement (e.g., oil, powder, or capsules) (Ahmad A, 2013). Higher doses did not significantly increase efficacy and were more likely to cause mild adverse effects. Black seed oil is one of the most common forms of supplementation. (Liao et al., 2022; Liao and Bai, 2022). The recommended dose is typically 500–1,000 mg per day, which can be divided into multiple doses. It is advised to take the oil after meals to reduce the risk of gastrointestinal discomfort. Black seed powder can be added to food or beverages (Zhang and Zhang, 2014; Boskabady et al., 2004). The recommended daily dose is one to two teaspoons (approximately 2–5 g), which can be mixed into water, juice, yogurt, or other foods. Due to the strong taste of the powder, it can be blended with honey or a sweetener to improve flavor. To see noticeable effects, it is recommended to use black seed supplements continuously for at least 2–4 weeks (Han and Chen, 2021). Based on the improvement of symptoms, the dosage and duration can be adjusted accordingly.

The administration methods included in the literature in this study included oral administration and nasal spray administration. Oral Administration (Nigella Sativa Oil or Capsules) generally considered safe when used in appropriate dosages (Moher et al., 2009). Common side effects are mild and may include digestive issues such as nausea or stomach upset. It is important to avoid excessive doses, as high amounts may cause adverse effects. Consulting a healthcare provider before starting is recommended, especially for individuals with underlying health conditions or those taking other medications (Otolaryngology, 2022). Nasal sprays are typically safe for most people and are effective in delivering the active compounds directly to the nasal passages. This method minimizes systemic absorption and targets the site of symptoms. Possible side effects may include mild nasal irritation or dryness. It is important to use a sterile preparation to avoid infections or contamination. While all methods can be safe when used correctly, nasal sprays are often considered the safest and most effective for treating allergic rhinitis because they deliver the active compounds directly to the affected area with minimal systemic effects (Zhang and Zhang, 2014; Boskabady et al., 2004). Nonetheless, it is important to follow dosage instructions and consult with a healthcare professional, especially for long-term use or in the presence of other health conditions.

Oral administration of Nigella sativa, either in capsule or oil form, was considered the safest route. This method minimized the risk of adverse effects compared to topical or inhalational routes, which were less commonly studied.

While our meta-analysis suggests that *Nigella* spp is effective in alleviating symptoms of allergic rhinitis, these findings are limited by a small number of studies and potential biases in trial design. Further large-scale, well-designed RCTs are needed to confirm these results.

## Data Availability

The original contributions presented in the study are included in the article/supplementary material, further inquiries can be directed to the corresponding author.

## Abbreviations

AR, allergic rhinitis; RCT, Randomized controlled tria; O, Odds ratio; CI, confidenceinterval; Nigella spp, Nigella sativa supplementation.

## References

[B1] Abdulghani Mohamed A. Mohamed Abdul S. Amina Hamed Ahmed A. (2012). “Evaluation of therapeutic efficacy of Nigella sativa (black seed) for treatment of allergic rhinitis,” in Allergic rhinitis. Editor Marek L. K. (Rijeka: IntechOpen). Ch. 12).

[B2] Ahmad A. Husain A. Mujeeb M. Khan S. A. Najmi A. K. Siddique N. A. (2013). A review on therapeutic potential of Nigella sativa: a miracle herb. Asian Pac J. Trop. Biomed. 3 (5), 337–352. PMID: 23646296; PMCID: PMC3642442. 10.1016/S2221-1691(13)60075-1 23646296 PMC3642442

[B3] Ali B. H. Blunden G. (2003). Pharmacological and toxicological properties of Nigella sativa. Phytother. Res. 17, 299–305. 10.1002/ptr.1309 12722128

[B4] Alsamarai A. M. Abdulsatar M. Ahmed Alobaidi A. H. (2014). Evaluation of topical black seed oil in the treatment of allergic rhinitis. Antiinflamm. Antiallergy Agents Med. Chem. 13, 75–82. 10.2174/18715230113129990014 23855426

[B5] Ansari M. Ahmad S. Khanum R. Akhtar M. (2009). Pharmacological investigation of protective effects of Nigella sativa oil in experimental diabetic neuropathy in rats. Indian J. Pharm. Educ. Res. 43, 166–176.

[B6] Bauchau V. Durham S. R. (2004). Prevalence and rate of diagnosis of allergic rhinitis in europe. Eur. Respir. J. 24, 758–764. 10.1183/09031936.04.00013904 15516669

[B7] Boskabady M. H. Kiani S. Jandaghi P. Ziaei T. Zarei A. (2004). Antitussive effect of Nigella sativa in Guinea pigs. Pak J. Med. Sci. 20, 224–228.

[B8] Brozek J. L. Bousquet J. Baena-Cagnani C. E. Bonini S. Canonica G. W. Casale T. B. (2010). Allergic rhinitis and its impact on asthma (aria) guidelines: 2010 revision. J. Allergy Clin. Immunol. 126, 466–476. 10.1016/j.jaci.2010.06.047 20816182

[B9] Chen L. (2020). Pharmacological effects and application status of the chemical components of the ethnic medicine black seed grass. Chin. Mod. Tradit. Chin. Med. 22, 985–990.

[B10] Gul R. Tansuker H. D. Cengiz A. B. Gul M. Tabaru A. Emre F. (2022). Effects of Nigella sativa oil on allergic rhinitis: an experimental animal study. Braz J. Otorhinolaryngol. 88 (Suppl. 5), S148–s155. 10.1016/j.bjorl.2022.09.003 36243604 PMC9801018

[B11] Han Y. Chen Z. (2021). Research progress on the composition, efficacy, and clinical application of black grass formulas. Chin. Mod. Appl. Pharm. 38, 252–256.

[B12] Han Y. Liao C. (2020). Research progress on the extraction process and pharmacological effects of chemical components from black grass. Chin. Mod. Appl. Pharm. 37, 1914–1920.

[B13] Higgins J. Green S. (2011). Cochrane handbook for systematic reviews of interventions. The Cochrane Collaboration. [updated march 2011].

[B14] Higgins J. P. Thompson S. G. (2002). Quantifying heterogeneity in a meta-analysis. Stat. Med. 21, 1539–1558. 10.1002/sim.1186 12111919

[B15] Huffman M. A. (2003). Animal self-medication and ethno-medicine: exploration and exploitation of the medicinal properties of plants. Proc. Nutr. Soc. 62, 371–381. 10.1079/pns2003257 14506884

[B16] Işik H. Cevikbaş A. Gürer U. S. Kiran B. Uresin Y. Rayaman P. (2010). Potential adjuvant effects of Nigella sativa seeds to improve specific immunotherapy in allergic rhinitis patients. Med. Princ. Pract. 19, 206–211. 10.1159/000285289 20357504

[B17] Jadad A. R. Moore R. A. Carroll D. Jenkinson C. Reynolds D. J. Gavaghan D. J. (1996). Assessing the quality of reports of randomized clinical trials: is blinding necessary? Control Clin. Trials 17, 1–12. 10.1016/0197-2456(95)00134-4 8721797

[B18] Kalus U. Pruss A. Bystron J. Jurecka M. Smekalova A. Lichius J. J. (2003). Effect of Nigella sativa (black seed) on subjective feeling in patients with allergic diseases. Phytother. Res. 17, 1209–1214. 10.1002/ptr.1356 14669258

[B19] Kjaergard L. L. Villumsen J. Gluud C. (2001). Reported methodologic quality and discrepancies between large and small randomized trials in meta-analyses. Ann. Intern Med. 135, 982–989. 10.7326/0003-4819-135-11-200112040-00010 11730399

[B20] Liao C. Bai S. Ulan Q. Gong S. Han Y. Lian H. (2022). Development and application of jia hei zhong cao formula for anti allergic rhinitis xilingol vocational college. Inn. Mong. Aut. Reg. 19, 292–296.

[B21] Moher D. Liberati A. Tetzlaff J. Altman D. G. PRISMA Group (2009). Preferred reporting items for systematic reviews and meta-analyses: the prisma statement. PLoS Med. 6, e1000097. 10.1371/journal.pmed.1000097 19621072 PMC2707599

[B22] Nikakhlagh S. Rahim F. Aryani F. H. Syahpoush A. Brougerdnya M. G. Saki N. (2011). Herbal treatment of allergic rhinitis: the use of Nigella sativa. Am. J. Otolaryngol. 32, 402–407. 10.1016/j.amjoto.2010.07.019 20947211

[B23] Otolaryngology T. N. G. o.t.E. C. o.t.C. J. o. (2022). Head and neck surgery, and the nasology group of the otolaryngology head and neck surgery society of the Chinese medical association Chinese guidelines for the diagnosis and treatment of allergic rhinitis (2022, revised edition). Chin. J. Otolaryngology Head Neck Surg. 57, 106–129.

[B24] Ridha C. M. (2023). Changes in total nasal symptoms score and quality of life after supplementation of Habbatussauda (Nigella sativa) in persistent Allergic Rhinitis. J. Kedokt. SYIAH KUALA, 1442–1026.

[B25] Tian Y. (2014). Development of compound black grass seed oil nasal drops. Jinan, China: master's disseration, Shandong University of Traditional Chinese Medicine].

[B26] Tungsukruthai P. Nootim P. Worakunphanich W. Tabtong N. (2018). Efficacy and safety of herbal steam bath in allergic rhinitis: a randomized controlled trial. J. Integr. Med. 16, 39–44. 10.1016/j.joim.2017.12.010 29397091

[B27] Wadiah S. B. (2005). The effects of Nigella sativa on allergic rhinitis. Saudi J. Otolaryngology Head Neck Surg. 7.

[B28] Wang X. Liu Y. Shi Y. Xu F. (2023). Anti-inflammatory and immunoregulatory effects of paeoniflorin and total glucosides of paeony. J. Immunol. 39, 120–127.

[B29] Zhang Y. Zhang L. (2014). Prevalence of allergic rhinitis in China. Allergy Asthma Immunol. Res. 6, 105–113. 10.4168/aair.2014.6.2.105 24587945 PMC3936037

[B30] Zhao J. Huang W. Zhang S. Xu J. Xue W. He B. (2020). Efficacy of glutathione for patients with cystic fibrosis: a meta-analysis of randomized-controlled studies. Am. J. Rhinol. Allergy 34, 115–121. 10.1177/1945892419878315 31550169

